# Turning toward mortality: yoga’s *savasana* as a salutogenic practice for engaging with death anxiety

**DOI:** 10.3389/fpubh.2026.1771782

**Published:** 2026-03-04

**Authors:** Lori Rubenstein Fazzio, Anne Pitman, Shelly Prosko

**Affiliations:** 1 Yoga Studies, Loyola Marymount University, Los Angeles, CA, United States; 2 Religious and Contemplative Studies, University of Ottawa and Centre for Health Innovation, Ottawa, ON, Canada; 3 PhysioYoga, Sylvan Lake, AB, Canada

**Keywords:** death anxiety, end-of-life care, mortality salience, salutogenesis, *savasana*, Sense of Coherence, yoga, yoga therapy

## Abstract

Death anxiety is prevalent across many modern cultures and is associated with significant psychological, social and economic costs, including avoidance of advance care planning and the overuse of life-prolonging medical interventions at the end of life. From a yogic perspective, this pervasive clinging to life reflects *abhinivesha*, one of the five *kleshas* (mental afflictions) described as a potent contributor to human suffering. This Perspective proposes a conceptual shift from pathogenic models focused primarily on life-extension and symptom reduction toward a salutogenic approach that emphasizes meaning-making and adaptive engagement with mortality across the lifespan. Antonovsky’s salutogenic framework highlights Sense of Coherence (SOC)—comprehensibility, manageability and meaningfulness—as a psychosocial resource that supports wellbeing even in the face of profound existential stressors. We suggest that cultivating conscious mortality awareness may strengthen SOC by supporting individuals to relate to death with greater understanding and existential coherence. Drawing on yoga philosophy, contemplative practice and public health scholarship, we propose *savasana* (corpse pose) as an embodied contemplative practice that may offer a structured experiential engagement with impermanence. Rather than positioning *savasana* as a treatment for death anxiety, we frame it as a salutogenic practice that can surface existential concerns and support reflective meaning-making over time. When practiced intentionally and upstream across the lifespan, *savasana* may provide an accessible, low-cost compassionate approach to facing death anxiety through embodied conscious awareness of our own mortality.

## 1 Introduction: the humanistic and economic cost of death anxiety

Death anxiety is prevalent across many modern cultures and is recognized as a significant contributor to human suffering (1–4). As a transdiagnostic construct, death anxiety is associated with numerous mental health disorders and is a key factor in avoidance of end-of-life planning (1, 4, 5). This may result in the extension of suffering due to unsuccessful, aggressive and costly medical interventions at the end of life (5, 6). Many people prefer to die at home surrounded by loved ones (7–9); however, the more the conversation around death is considered taboo, the less likely the person may be to engage in end-of-life care discussions. This may delay access to timely palliative care, thereby reducing quality of life during one’s final days (5, 10). Although advance directives aim to preserve individual autonomy at the end of life, their implementation remains variable within complex healthcare systems. While multiple studies across varied cultures indicate the majority of people would prefer palliative care (6, 11), not everybody receives it. Due to socio-cultural-political-economic circumstances, most die either unsupported at home without comfort care (12) or die in the ICU following expensive and futile life-prolonging interventions that may impose significant humanistic and economic costs on individuals, families and public health systems (6, 13, 14). Within this context, there is need for approaches that address death anxiety upstream and cultivate adaptive ways of relating to mortality across the lifespan.

## 2 Insights into death anxiety

Throughout history, the fear of death has been documented in art, literature, religion and science. Many macro-level determinants contribute to one’s relationship to death. It is beyond the scope of this article to provide a comprehensive history of death anxiety; however, we highlight some key factors relevant to the death phobia prevalent in modern medicalized societies. For example, prior to the advent of germ theory that was developed in the mid-19th century by Louis Pasteur and Robert Koch, death in Western Christian-based culture was marked by a ceremony that occurred in the home with the dying person “lying in their deathbed” surrounded by family, including children (15). French social historian Philippe Ariès referred to this as a “Tamed Death” focused on acceptance, rituals and spiritual transition. Ariès theorizes that our relationship to death gradually evolved from a communal, familiar and accepted part of life to being hidden, medicalized and avoided (15, 16).

Germ theory catalyzed a reframing of death as a biological failure that could be prevented through medical management, typically via hospitalization. Abraham Flexner’s report of 1910 further institutionalized dying, relocating end-of-life care from a spiritual transition at home to what Ariès termed a “Forbidden Death”: an expensive, medically managed clinical event that often results in the prolonging of life and suffering, typically with the patient dying alone and connected to machines (14, 17). Ariès cautions that this shift from acceptance of mortality to a culture of death phobia severs our connection to perhaps the most profound opportunity for meaning and dying well (15). Terror Management Theory, developed by Greenberg, Pyszczynski and Solomon in the 1980s (18), posits that fear of death can be a driving force behind many health behaviors (1, 19) including avoidance of advance care planning and overuse of aggressive life-extending medical interventions (5). These dynamics underscore the importance of approaches that do not merely suppress anxiety but foster healthier relationships with mortality.

### 2.1 Understanding the fear of death: *abhinivesha* and the clinging to life

The pervasive modern cultural phenomenon of death avoidance reflects the yogic concept of *abhinivesha*, one of the five *kleshas* or mental afflictions that are understood to be the root causes of suffering (20). While commonly translated as “fear of death,” the concept traditionally refers to clinging to the manifest form of life (3). The fourth century Sage Vyasa’s translation of Patanjali’s *Yoga Sutra* elucidates the concept of *abhinivesha*: “May I not cease to exist. May I live on” (20). A contemporary example of this clinging to life includes practices of embalming and cosmetology that attempt to preserve a life-like resemblance for viewing during a funeral.

Conversely, the cultivation of mindful mortality salience, or awareness of one’s own inevitable death, prompts individuals toward adaptive coping mechanisms including finding meaning and purpose, reducing anxiety and increasing emotional resilience. For example, the Buddhist contemplative practice of *maranasati* (mindfulness of death) invites acceptance and equanimity around death through a systematic reflection on the inevitability of death (1). While research is limited, modern mindfulness practices inspired by these teachings have been shown to reduce death anxiety (21) and increase Sense of Coherence (SOC), a core concept of salutogenesis, as outlined below.

## 3 Salutogenesis and yoga

Aaron Antonovsky, a medical sociologist, challenged the pathogenic model of medicine in the 1970s by proposing that the field shift from the question of “what causes disease?” to the study of “what creates health?” Antonovsky frames health as not merely the absence of disease, but as the capacity to cope with stress, find coherence and create meaning amidst life’s challenges (22). This emphasis on the inherent human need for meaning-making finds a profound parallel in the work of philosopher and psychiatrist Viktor Frankl. Frankl, a Holocaust survivor, identified a sense of existential purpose as fundamental to enduring suffering, making his perspective highly relevant to the concept of self-transcendence central to this paper. Within his framework of logotherapy, Frankl (23) defines self-transcendence as the human tendency to reach out beyond oneself and find meaning, love and service in life (24). Multiple studies affirm self-transcendence as being positively associated with the ability to cope effectively with death anxiety and improved wellbeing at the end of life (25–28).

Antonovsky’s salutogenic model of medicine posits that SOC, which includes comprehensibility, manageability and meaningfulness, is the key to health until one’s very last breath (29). If we apply this framework to helping people face death, it may include:

- *Comprehensibility:* Support people in better understanding the dying process to reduce fear and confusion.
- *Manageability:* Support self-agency, accessing inner and outer resources and practicing accessible pain self-management techniques.
- *Meaningfulness:* Support contemplation, prayer and meditation, life review, legacy projects, forgiveness practices and final conversations.

The demystification of death could begin upstream long before one is at the end of life. Yoga, with its philosophy and practices, inherently aligns with this salutogenic framework. Yoga employs the concepts of *samadhi* (transcendence), *vairagya* (detachment or letting go), *vichara* (self-awareness), *svadhyaya* (understanding self through studying yoga) and *sadhana* (personal practice) as foundational approaches throughout life that are of paramount importance when facing death (30, 31). These philosophical pillars align with the salutogenic emphasis on cultivating internal resources to navigate existential distress.

## 4 Yoga therapy in end-of-life and palliative care

Yoga practices that support mind, body and spiritual health are increasingly being integrated into modern healthcare (32) and holistic palliative practices (31, 33–40). Yoga therapy, an individualized therapeutic approach, adapts yoga philosophy and practices to the changing physical, mental, emotional and spiritual needs of the living and dying person and is considered valuable as a non-invasive, non-pharmacologic complement to integrative palliative care (30, 39–41). Yoga therapists, through co-regulated compassionate inquiry, help people engage in their decisions and care, cultivating inner and outer resources for support and to prepare for their dying days. Yoga studies highlight various adaptations of yoga practices for integrative palliative care including mindfulness, gentle physical movement, regulated breathing techniques, subtle hand gestures, meditative practices, conscious yogic sleep and chanting or singing (30, 34, 37, 38, 40).

End-of-life research focusing on yoga, mindfulness and meditation notes that these practices can help manage anxiety and depressive symptoms, appetite, fatigue and insomnia (42). They also may improve perceived stress (43), inform an accepting attitude toward pain (44), inspire confidence and self-efficacy (38), foster compassion and empathy (45) and cultivate a nonjudgmental attitude to whatever arises in the present moment (46). Spiritual support, when facing death, has been shown to improve quality of life, spiritual wellbeing, insight and inner peace for terminally ill patients (42, 47–51). Corpuz (52) proposed that integrating spirituality and mindfulness into palliative care may lead to profound benefits for both patients and caregivers, offering comfort, solace and a sense of purpose in the face of mortality. While fear of death is common at life’s end, neurophysiological research suggests that death avoidance is not an innate neural circuit or genetically determined response, but can be positively informed by meditation on the impermanence of the self, which can induce neural reorganization (53). Practices that help people face their fear of death can reduce death anxiety (1, 53–55) and may improve advance care planning outcomes (50).

## 5 *Savasana* as a salutogenic practice

**Figure 1:**
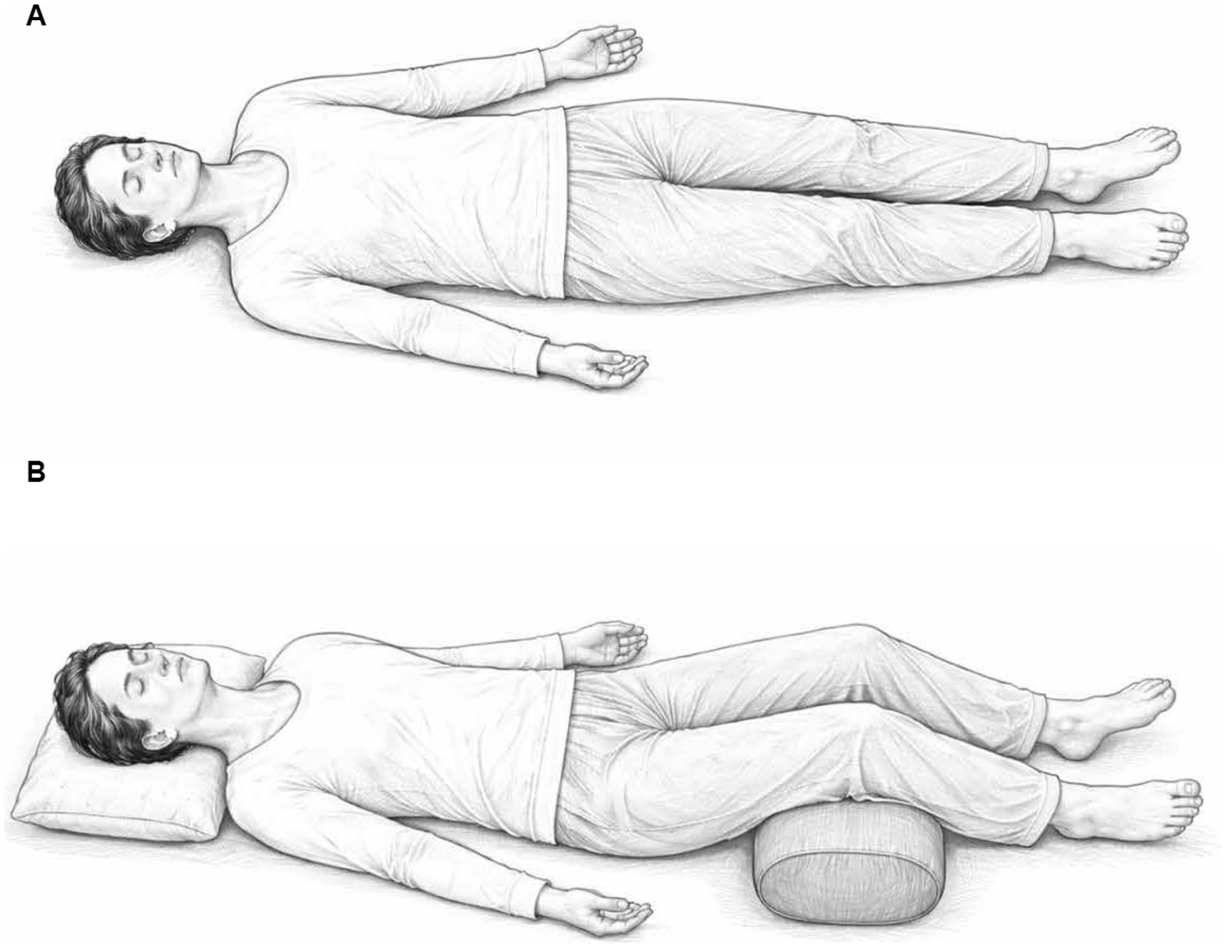
**(A)** Savasana (Corpse pose). Arms and legs can be close together or slightly away from the body as shown. **(B)** Savasana (Corpse pose) Variation: Savasana can be adapted for comfort and accessibility with the use of supportive pillows and bolster as shown above.

One early and seminal paper on end-of-life yoga therapy proposed an eminent yoga practice—widely known from ancient yoga texts and still resonant in modern times—that is surprisingly underutilized in death preparation yet perhaps the most obvious choice: *savasana* (30). *Savasana*, translated to “corpse pose” in English, is a universally practiced and widely recognized yoga posture that completes yoga group classes worldwide (Figure 1). This pose, however, is often solely equated with relaxation and rejuvenation without mention of its essence, which is to lie still as a corpse. In what may reflect culturally embedded death phobia, the word *savasana* is rarely translated, even when all other yoga practices are referred to in English. Rarely, in modern times, is mortality or dying mentioned in corpse pose, nor is it recognized that, rather than being morbid, *savasana* practice could both guide death education and revitalize a love of life.

Yoga is widely embraced and practiced by millions of practitioners across diverse communities globally (56). With deep respect to the ancient roots of Yoga, we propose that *savasana* (corpse pose)—which we suggest as an embodied practice of contemplating death—may offer an accessible and cost-effective means of improving mortality salience and possibly, through continued practice, reframing today’s cultural obsession with prolonging life. Importantly, we frame *savasana* not as a panacea for death anxiety, but as a salient component of a salutogenic approach to facing mortality with conscious awareness.

### 5.1 *Savasana:* from restorative posture to contemplative engagement with mortality

The following description is offered as a phenomenological and pedagogical illustration rather than a literal, universal or prescriptive account of *savasana* practice.

A mortality-awareness *savasana* practice typically begins with the participant lying comfortably in a supine position and being guided to release muscular tension, regulate breathing and cultivate a subjective sense of safety and grounding. They are encouraged to remain oriented to present-moment bodily sensations and to regulate the depth of engagement according to comfort and readiness. The facilitator may then invite gentle contemplative inquiry by asking the participant to imagine that this resting moment represents the final moments of life, not as a literal rehearsal of dying, but as a symbolic exploration of impermanence and letting go. Attention may be sequentially directed toward sensory awareness such as noticing breath, sound, touch or taste as if this were the final experience of that sense. This phenomenological inquiry invites awareness of transience without requiring adherence to any specific metaphysical belief system. The practice may also include reflective prompts inviting the temporary release of attachment to roles, identities, achievements and habitual self-concepts. In yogic terms, this can be understood as experiential inquiry into dis-identification from the manifest aspects of experience (*prakriti*), allowing attention to rest in simple awareness and, ultimately, toward recognizing what yoga philosophy describes as our pure, unchanging nature (*purusha*) (20). Unlike conventional *savasana*, which is commonly framed primarily as rest, recovery and physiological integration, mortality-aware *savasana* intentionally introduces mindful and intentional engagement with impermanence and uncertainty. Practiced gradually, this approach may support increased tolerance of existential discomfort, greater familiarity with themes of loss and change and expanded capacity to remain present with mortality-related thoughts and emotions. This practice is not intended to replace psychological, spiritual or medical care, but rather to offer a contemplative and embodied framework for cultivating reflective awareness of mortality within a broader salutogenic orientation that seeks to enhance comprehensibility, manageability and meaningfulness.

Across canonical *hatha yoga* sources—including the *Hathapradipika, Hatharatnavali*, and *Gheraṇda Samhita*—*savasana* is consistently presented as a posture of stillness, recuperation and mental settling following exertion, with little elaboration regarding existential or contemplative intent (57–59). Taken together, these texts suggest that *savasana* has historically been understood as a posture of rest, mental settling and recovery—one that prepares the practitioner for deeper yogic processes but does not, in itself, prescribe a specific contemplative orientation. One notable exception appears in the *Dattatreya Yogasastra*, where lying supine “like a corpse” is mentioned in the context of *layayoga*, the dissolution of mental activity (60). While this text does not name the practice as *savasana*, the description aligns with that of other *hatha yoga* texts with minimal instruction, and the emphasis on the quieting or dissolution of the mind. While the goal in this context is transcendence, contemplation of one’s biological death is not explicitly stated; although, it could be inferred given the instructions are to lie like a corpse.

Building on this traditional foundation, we propose a contemporary framing of *savasana* that remains consonant with yoga philosophy while extending its application within a salutogenic and public health context. Rather than redefining the posture, we suggest that a mortality-awareness *savasana* may be approached intentionally as an embodied contemplative practice that invites awareness of impermanence. This proposal does not rest solely on the English translation “corpse pose,” but on the posture’s defining characteristics: physical stillness, cessation of effort and the quieting of sensory and mental activity.

Rather than a tool for suppression of symptoms, *savasana* practice provides an opportunity to practice self-awareness—a concept Bhide et al. (61) identify as central to yoga’s therapeutic potential. This invites contemplative engagement with mortality, whereby the practitioner cultivates a capacity to observe their internal experience as it is. The cyclical movement into stillness and back into wakeful participation in life supports experiential familiarity with impermanence without overwhelming the practitioner and may foster adaptive engagement with mortality that supports comprehensibility, manageability and meaningfulness across the lifespan.

### 5.2 Upstream *savasana* for dying well

The value of *savasana* at the end of life and in palliative care has considerable potential. Beyond its application in end-of-life contexts, we propose that in cultures and healthcare systems where death is often ignored or denied until the final stages—frequently with burdensome consequences—a regular practice of *savasana* upstream across the lifespan may help cultivate experiential familiarity with impermanence, foster personal and community resources, support a sense of agency in how individuals relate to dying and cultivate a coherent response to mortality.

### 5.3 *Savasana* and the dimensions of Sense of Coherence

We propose integrating key elements of the salutogenic concept of SOC with the embodied practice of *savasana*, detailing their alignment below:

#### 5.3.1 Comprehensibility

*Savasana* offers the practitioner a regular practice of facing their mortality and engaging with the mystery of death. Learning to witness the concept of one’s own impermanence can be explored by engaging in a conscious, experiential practice of the dying process through embodied inquiry of kinesthetic awareness, physical sensations, breathing, emotions, thoughts and beliefs around death. This may inspire the practitioner to engage in valuable conversations about preparing for their death practically, emotionally and spiritually. Importantly, the yoga professional guiding the practice must allow for and respect the practitioner’s own spiritual and religious beliefs, rather than imposing any aspects of yoga philosophy that may not align with the practitioner’s views.

#### 5.3.2 Manageability

When the comprehensibility of death is supported, *savasana* practice may help each person develop agency and the ongoing resources to remember themselves as mortal and death as inevitable. Through various yogic visualization practices, a person can be guided to plan how they might want to die (people, place and rituals). Individuals can also be guided to explore and engage in techniques to modulate or cope with pain. Concurrently, developing the skill of “letting go” can acknowledge fear, calm panic and help to release the strong grip of the need to control.

#### 5.3.3 Meaningfulness

Contemporarily, death is often seen as a “failure” and is frequently viewed as “giving up” or having “failed in the battle.” The dying individual, no longer perceived as contributing to society, can feel lonely and abandoned, as though their life and death lack meaning.

A consistent and compassionate practice of contemplating death via *savasana* offers an opportunity to cultivate greater awareness and acceptance of one’s mortality. The practice may foster a recognition of the significance and purpose of the person’s life and death. It may encourage deep, meaning-centered reflections: reviewing one’s life, considering one’s legacy and inspiring satisfying final conversations. Additionally, facing death more consciously can provide a living example for younger generations contending with their own death anxiety. Corpse pose, when practiced with intention, contemplation and compassion, can facilitate a sense of meaning-making that may support both an individual’s experience of dying and the community from which they die.

## 6 Future considerations and directions

### 6.1 Research

Future research could investigate quantitative and qualitative outcomes, economic considerations related to healthcare costs and the efficacy of a mortality-salient *savasana* practice’s impact on death anxiety and overall wellbeing across the lifespan.

### 6.2 Education and training

Professional yoga education may benefit from integrating Antonovsky’s salutogenic model with the philosophy and practice of *savasana*, incorporating concepts of death, dying, *abhinivesha* and SOC.

### 6.3 Therapeutic and community implementation for public health

To address upstream engagement, a focus on curating community resources is recommended, including workshops designed to explore SOC elements, facilitate open discussion of death and dying and offer inclusive *savasana* practice in both therapeutic and community group class settings.

### 6.4 Cultural and ethical considerations

Given the broad public health relevance, it is vital to develop *savasana* guidelines tailored for diverse cultural and religious populations with varying beliefs that could be implemented across various settings including yoga group classes and one-on-one yoga therapy.

## 7 Conclusion

Due to the extensive social, cultural, psychological and physiological consequences of death anxiety, as well as its substantial burden on public health systems, there is a need for feasible, cost-effective and compassionate approaches to engage with death anxiety and enhance mortality salience within modern cultures that have medicalized and vilified death. We contend that a conscious, mortality-awareness practice of *savasana* offers an accessible means for individuals to turn toward death, thereby cultivating the Sense of Coherence (SOC) necessary to navigate life’s ultimate uncertainty. By engaging with the inevitability of our death, *savasana* has the potential to ease the suffering that accompanies death anxiety, supporting individuals not only in approaching death with greater awareness but also modeling an engaged relationship with mortality for future generations.
